# An Agenda-Based Routing Protocol in Delay Tolerant Mobile Sensor Networks

**DOI:** 10.3390/s101109564

**Published:** 2010-10-28

**Authors:** Xiao-Min Wang, Jin-Qi Zhu, Ming Liu, Hai-Gang Gong

**Affiliations:** School of Computer Science and Engineering, University of Electronic Science and Technology of Chengdu, 610054, China; E-Mails: xiaomin.wang@126.com (X.-M.W.); jingpei719@163.com (J.-Q.Z.); hggong@uestc.edu.cn (H.-G.G.)

**Keywords:** DTMSN, social, mobility model, routing

## Abstract

Routing in delay tolerant mobile sensor networks (DTMSNs) is challenging due to the networks’ intermittent connectivity. Most existing routing protocols for DTMSNs use simplistic random mobility models for algorithm design and performance evaluation. In the real world, however, due to the unique characteristics of human mobility, currently existing random mobility models may not work well in environments where mobile sensor units are carried (such as DTMSNs). Taking a person’s social activities into consideration, in this paper, we seek to improve DTMSN routing in terms of social structure and propose an agenda based routing protocol (ARP). In ARP, humans are classified based on their agendas and data transmission is made according to sensor nodes’ transmission rankings. The effectiveness of ARP is demonstrated through comprehensive simulation studies.

## 1. Introduction

Advances in wireless communications and integrated circuits have enabled the development of small, smart and inexpensive wireless sensor devices. We envision that in the very near future these sensor nodes will be embedded into a multitude of human-carried devices. A large number of these mobile devices can be dynamically networked together and form wireless sensor networks (WSNs). Due to unpredictable human mobility, topology changes, limited transmission range of sensor nodes and so on, this type of network often exhibits extremely low and intermittent connectivity. Intermittent connectivity is a key characteristic of delay tolerant mobile sensor networks (DTMSNs) [1,2] while the network formed by mobile devices is one popular communication paradigm of DTMSNs. Data gathering is one of the major functions of WSNs.

Data gathering approaches in the traditional WSNs usually rely on a large number of densely deployed sensor nodes with short range radio to form a well connected end-to-end network. Sensors in the network collaborate with each other to collect the target data information and transmit them to the sink nodes [3]. Such approaches, however, may not work effectively in DTMSNs due to intermittent network connectivity and sensor node mobility. Thus, routing in DTMSNs is a quite challenging problem. Meanwhile, currently, most DTMSN routing schemes just use simplistic random models for algorithm design and performance evaluation. e.g., the most commonly used mobility model is random waypoint [4] in which all nodes move randomly and their moving speed and direction are drawn from the same distributions. However, due to the differences in human behavior, the random waypoint model cannot reflect realistic human mobility characteristics [5]. Therefore, previous DTMSN routing protocols would not work well in mobile human applications and new routing schemes are required.

There have been a number of routing techniques proposed for human-carried scenarios in disconnected delay tolerant networks. In [6], the authors imagine that each node in the network has a label telling others about its affiliation. A node only chooses to forward messages to destinations, or to next-hop nodes belonging to the same group (same label) as the destinations. It was demonstrated that LABEL significantly improves forwarding efficiency over oblivious forwarding using their one dataset, but it lacks a mechanism to move messages away from the source when the destinations are socially far away. Chaintreau *et al*. [7] presented an analytical foundation on the impact of human mobility on the design of opportunistic forwarding algorithms based on six real human mobility traces from four different research groups. They established the inter-contact intervals, and contact durations for a wide range of typical human mobility patterns and for a variety of radio devices. Critically, it was shown that stateless forwarding schemes would not provide a bounded expected mean delivery latency across such systems.

In this paper, we present an agenda based routing protocol, ARP, which uses peoples’ agendas and their social relationships to carry out routing. Since people have varying social roles in the real world, some people may more popular and interact with sink nodes more often than others in the network. Thus in ARP, humans are ranked based on their popularity and routing decisions are made according to two key variables, which are transmission rankings and communication probabilities of sensor nodes, respectively. The first part is to route messages to nodes whose transmission rankings are higher than the current node, while the second part is to identify node’s encounter time with sink nodes and to use them as relays. Moreover, ARP also employs the message survival time to decide a message’s transmission or dropping to minimize transmission overhead. We evaluate the effectiveness of ARP by simulations and compare its performance with previous schemes under a mobility model which combines both the social activities and the geographic movements. Simulation results show that ARP achieves a relatively longer network lifetime and higher message delivery ratio with lower transmission overhead and data delivery delay than both flooding and LABEL schemes.

The main contributions of this paper are to answer these questions:

1. How does the variation in node transmission ranking help us in data forwarding?
2. Why and how to design the message queue management scheme? Since the buffer queue of a sensor node is limited, queue management is another challenge for effective data delivering.
3. How well does ARP work? How does it compare to other routing protocols in our network model that reflect the characteristics of human mobility?

The rest of the paper is organized as follows: Section 2 discusses related works. Section 3 discusses the motivation and the mobility model used in our paper. Section 4 introduces ARP. Section 5 shows the effectiveness of ARP via simulation. Section 6 concludes the paper.

## 2. Related Work

Routing strategies under delay tolerant scenarios have been explored by a number of research groups. The most basic and simple protocol is direct transmission, where data would only be delivered when sensors are in direct proximity of the sinks. That protocol has very little communication overhead, given that messages are only sent directly from the source sensor node to the sink node. However, depending on how frequently sensor nodes meet the sink nodes, the delivery of the data is very poor and the delivery ratio might be very low. This is particularly true if the sink nodes are very few and spread out. Another basic protocol is the flooding protocol [8], where a sensor always broadcasts the data messages in its buffer to all the nearby sensors, which receive the data messages, keep them in the buffer queue, and rebroadcast them. If the queue is large enough, the flooding protocol could achieve a low data delivery delay and high delivery ratio at the cost of more traffic overhead and energy consumption. However, the buffer size of sensor is usually limited, which results in many messages being dropped and frequent need for retransmission.

The Shared Wireless Info-Station (SWIM) system is proposed in [9] for gathering information from radio-tagged whales, assuming that randomly moving sensors have the same probability of meeting the sink, and thus a sensor node needs only to distribute a number of copies of a packet to other nodes so as to attain the desired data delivery ratio. By considering the characteristics of DTMSNs, the ZebraNet project [10] proposes a history-based approach for routing. The routing decision here is made according to a sensor node’s past success rate in transmitting data packets to the sink nodes directly. However, the data delivery ratio here is very low.

The Data MULE project uses mobile nodes to collect data from sensors which are then delivered to a base station [11]. A mobile entity called data mule receives data from the nearby static sensors, temporarily store them, and drops off the data at the access points. However, they do not consider opportunistic forwarding between the mobile nodes. Spyropoulos *et al*. [12] used a combination of random walk and utility-based forwarding. Random walk is used until a node with a sufficiently high utility metric is found after which the utility metric is used to route to the destination node. Leguay *et al.* [13] presented a virtual coordinate system where the node coordinates are composed of a set of probabilities, each representing the chance that a node will be found in a specific location. This information is then used to compute the best available route.

Other endeavors aiming to enhance the performance of DTMSN routing include [14,15]. In [14], a replication-based efficient data delivery (RED) protocol is presented. RED uses a history-based method like ZebraNet to calculate the delivery probabilities of sensor nodes. When message transmission occurs, the delivery probability of the source sensor increases; contrarily, if there is no transmission during a given interval, the delivery probability would decrease. However, in DTMSN scenarios where nodes are sparse and intermittently connected, it often takes a comparatively long time for a node to meet another which has a higher delivery probability. Therefore the updating frequency of the delivery probability in RED is usually low, and there may exist sensors which are far away from the sink but still have high delivery probabilities. Thus the history-based method is not effective and cannot denote the actual ability that a node delivers data to sink nodes. In addition, the message management of propagating many small messages in the network may incur further processing overhead and inefficiency of bandwidth utilization. In [15], the authors propose a FAD protocol to increase the data delivery ratio in DTMSN. Besides using the same delivery probability calculation method as RED, FAD further discusses how the replication of the data over the sensor network can be constrained using a fault tolerance value associated to each data message. That protocol still has quite a high overhead.

In the recent years, social structures have been used to help forwarding in intermittently connected networks. For example, the authors in [16] consider the communities formed by nodes in the network for control flooding. They assume that nodes mainly remain inside their community and sometimes visit others. To route a message to a destination, a node may transfer that message to a node that belongs to the same community as the destination. Their work provides a theoretical hypothesis for community based routing, but there is not yet any empirical evaluation. Daly *et al*. [17] presented the SimBet routing metric which is comprised of both a node’s centrality and its social similarity. If the destination node is unknown to the sending node or its contacts in SimBet, the message is routed to a structurally more central node where the potential of finding a suitable carrier is dramatically increased. Simulation results demonstrate that SimBet routing comes close in terms of performance to that of epidemic protocol [18], without the additional overhead of redundantly forwarding the message.

In [19], real movement traces in a certain region have been gathered and analyzed for predicting characteristics of human mobility. These characteristics can be used as a complement of data delivery schemes. However, due to the limited region size in trace gathering, whether these traces can reflect the true human mobility characteristics need to be verified.

## 3. Network Model and Problem Statement

### 3.1. Network Model

**Figure 1.**
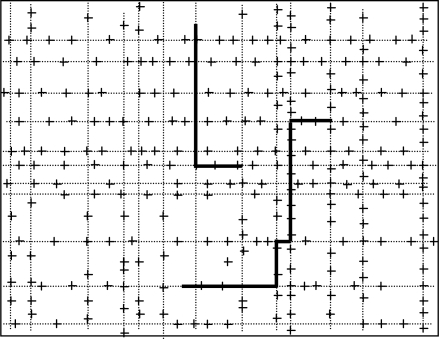
Traveling pattern of the moving node in our network model.

We assume initially all the sensor nodes are randomly deployed in an urban area that consists of streets running east-west and avenues running south-north. Distances between neighboring streets (or avenues) are randomly chosen, they are not equally reparted. The lengths of streets and avenues are also randomly chosen within a certain range. Furthermore, from south to north, streets are numbered 0,1,2,..., and from west to east, avenues are numbered 0,1,2,...., as can be seen from Figure 1. Additionally, the addresses which are used to locate the places where people will stay for the defined activities are randomly distributed on the roads. Each address is associated with the activities people can do at the place, e.g., a restaurant, an office, *etc.* All the sensor nodes are homogeneous and have a unique ID number. The maximum transmission range of all the nodes is fixed to R. A number of static sink nodes are randomly deployed in the network.

The mobility of all sensor node is assumed to follow the agenda based mobility model depicted in [20] where each sensor node carries a unique agenda that describes its whole days journey. Each item of the agenda indicates when and where the node will be. A node moves only according to its agenda and it moves from the current location towards the next one in its agenda by using Dijkstra’s Algorithm. Besides, each sensor node has a home from where the node always starts and to where it returns before midnight.

### 3.2. Problem Statement

Mobility modeling is important in wireless and mobile networking research. Different scenarios require different mobility models. Hence being able to design a mobility model which reflects real world characteristics has been a critical requirement. Research results also show that mobility models influence the performance of protocols significantly [20]. It is known that humans’ motion behavior has the following unique characteristics: (1) Location preferences, that is, some people are more likely to go to certain locations, and then interact more frequently with some certain nodes than others. (2) Time-variant mobility. Humans’ activity changes with respect to time. Different activities occur at different times and locations. However, most widely used models are very simplistic, and they generate traces that show properties very different from those extracted from real scenarios.

For example, the random waypoint mobility model and the random direction model are the two most widely used mobility models. In the first model, nodes randomly select destination, speed, and pause time at the destination. In the random direction model, nodes randomly select directions. In these two models, nodes move freely and all nodes generate homogeneous behavior. They do not reflect social connections among mobile users nor the possible influence of the connections on motion behavior. However, the network model used in our paper emphasizes the importance of humans’ social roles [20], and it combines both the social activities and the geographic movements. Table 1 summarizes the characteristics of some mobility models. Based on these analyses, we propose a novel routing protocol called ARP that use peoples’ agendas to carry out routing.

## 4. ARP Routing Protocol Design

### 4.1. Data Transmission

People act in different social roles and differ in their popularity. e.g., some people are more popular than others in the society. This should also be true for sensor nodes in the network. We only employ heterogeneous popularity to help designing an efficient routing strategy. In ARP, data transmission decisions are made according to two parameters. The first one the called the transmission rankings, which indicate the likelihood that sensor nodes will communicate with the sink nodes. Generally, the more likely a node is to communicate with sink nodes, the higher the transmission ranking attached to it. The second one is the communication probability that is used to identify a sensor’s communication strength with sink nodes at different time periods of a day.

First we look at how to define the transmission ranking value of sensor nodes. Without loss of generality, we let *Pi* denote the transmission ranking of sensor *i*. Due to peoples’ regular movement patterns, their social relationships vary much more slowly, and thus their transmission rankings are relatively fixed. We assume each sensor node needs to maintain a table that contains a list of transmission rankings of encountered nodes and its corresponding cumulative contact duration with nodes attached to the same transmission ranking. Let *Fi* denote the contact table for sensor node *i*, the process of determining *Pi* can be described as follows.

- *Pi* is initialized to be zero.
- If one of the addresses associated with the agenda for sensor node *i* comes within the transmission range of the sink node, no matter where the node is, *Pi* is set to Δ (where Δ is a large integer used to represents the highest ranking). This is because sensor node *i* can communicate directly with the sink nodes sooner or later in such case.
- If the above case can not be held, then when a mobile node *i* interacts with a node *k*, if the transmission ranking of node *k* is not included in *Fi*, one item will be added in *Fi* to record the contact information: the transmission ranking of node *k* and their contact duration. Otherwise, the corresponding cumulative contact durations depicted in *Fi* will be updated in a timely fashion.
- If and only if the total contact duration count of node *i* and sensor nodes possessing higher transmission rankings than node *i* exceeds a certain threshold *Th* (where *Th* is the upgrading threshold which we will vary in this paper to see its impact on the network performance), *Pi* is increased.
- When node *i* cannot contact with nodes with higher transmission rankings than itself for a certain period of time (e.g., λ), the transmission ranking of node *i* will be decreased accordingly.

**Figure 2.**
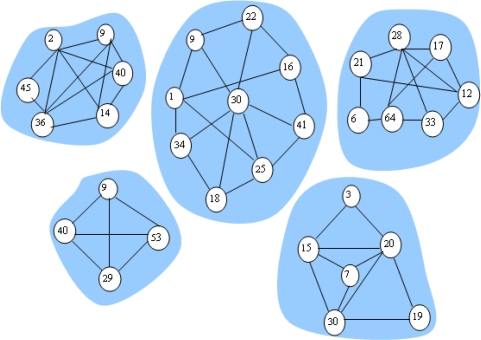
Community structure.

We define the community as formed by sensor nodes with the same transmission ranking. Thus, the social network can be partitioned into a set of communities through the aforementioned method. Figure 2 depicts a network consisting of five communities. Furthermore, for nodes with the highest transmission ranking, since they contact with sinks at different times, their communication probabilities vary with time. We let *Tp* denote the current time and let the communication probability of node *m* with the sink node *n* be *Ymn*, when *Tm* > *Tp*, we have:

(1) $$\mathrm{Ymn}\;=\;1\;-\frac{\left(\mathrm{Tmn}\;-\;\mathrm{Tp}\right)}{24}$$

where *Tmn* denotes the encounter time between node *m* and the sink node *n* (*n* = 1,2,3…). Otherwise, *Ymn* = 0. The communication probability of sensor node *m* is set to the largest value of *Ymn*, e.g., the current time is 9:00 AM, the encounter time between node *m* and the two sink nodes *a* and *b* in the network are 10:15 AM, and 13:00 PM, respectively. *Ym* equals 0.948 according to Formula (1).

**Figure 3.**
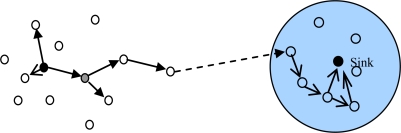
Sketch of the routing algorithm.

Data forwarding is carried out as follows. We also consider the sensor *i*, which has a message in the data queue ready for transmission and is moving into the communication range of a set of Z’ sensors. Let ∑ = {Ψz | 1 ≤ z ≤ Z’} represent the *Z’* nodes. Sensor *i* first learn its transmission ranking via simple handshaking messages and then replicates the message in its queue to a subset of the *Z’* sensors, which have higher transmission rankings than *Pi*. When the message reaches the node with the highest transmission ranking, the communication probability metric is used instead of the transmission ranking metric and the message continues to deliver to nodes with communication probabilities higher than the current node until the destination is reached. This method works in a distributed way, it does not require every sensor node to know the ranking of all other nodes and sensor nodes in the network just need to be able to compare transmission rankings or communication probabilities with the node encountered. Figure 3 illustrates the ARP algorithms.

### 4.2. Queue Management

In opportunistic networks like DTMSNs, multiple copies of messages may be generated and buffered by different sensors, resulting in redundancy. In order to reduce cost, the queue management scheme is employed. The main idea of our queue management is to employ both the message survival time and priority to signify the importance of a given message.

#### 4.2.1. Message’s Survival Time

We assume each data message has a field to record its survival time. When a message is generated, its survival time is initialized to be zero. In order to update the survival time, each sensor maintains a timer. Once the timer expires, the stored message’s survival time are increased. Moreover, let’s consider a sensor, which is transmitting a data message j to a nearby sensor node Ψz. Since the propagation time between two adjacent nodes with short distance could be ignored, thus the receiver would insert the message to its own queue directly without any modification to the value of survival time. If a source message has transmitted to its next hop and it is inserted into node’s queue again, its survival time is also assumed to be equal to the value before transmitting.

#### 4.2.2. Message’s Priority

Messages in the buffer queue come from three sources: (a) when the sensor acquires data from its sensing unit, it creates a data message and inserts it into the queue; (b) when the sensor receives a data message from other sensors, the message is inserted into the data queue; (c) after the sensor sends out a data message to a non-sink node, it may insert the message again if the message is created by the source sensor node itself, because the message is not guaranteed to be delivered to the sink node. Messages belonging to the first class are set to have the highest priority level, while messages from the second and the third class are prescribed to have the middle and the lowest priority level accordingly.

#### 4.2.3. The Implementation of the Queue Management Scheme

Our queue management scheme is based on both the priority and the survival time of messages. At first, messages in the queue are arranged with a decreasing order of their priority. Since we believe that the message with higher priority level is more important, it should be transmitted with a higher priority. Furthermore, for messages with the same priority level, priority should be given to those messages that have smaller survival times. A message is dropped in one of the following two occasions: once the survival time of a message in the process of updating exceeds the network’s delay tolerant threshold, the message is dropped. Second, if the queue is full when a message arrives, its priority level is compared with the message at the end of the queue. If the new message has a lower priority level, it is dropped. Otherwise, the message at the end of the queue is dropped, and the new message is inserted into the queue at appropriate position according to both its priority level and survival time. This is to reduce network energy consumption, given that the message either has been delivered to the sink node with a high probability by other sensors or has been invalid in our application.

## 5. Simulation Study

### 5.1. Simulation Environment

In our experimental environment, we assume the simulation area is a 400 m × 400 m region with a total of 60 addresses on the map. More specifically, 33.3% addresses are homes, 25% addresses are schools, 25% addresses are offices and 16.7% addresses are other types of places, e.g., restaurants or shops. In our simulation scenario, there are three types of nodes: the first type is students whose first activity must be go to school; the second type is workers whose first activity must be go to work and the third type is others, whose first activity could be any of activities except going to work and going to school. The time of the first activity for all the nodes is between 6 AM and 8 AM. Furthermore, every node goes back to its home before midnight. The number of activities of any kind of nodes is between 1 and 10.

We assume the network bandwidth is 5 kbps, and the simulation runs for three days. Other simulation parameters and their default values are summarized in Table 3. In particular, we assume that in flooding protocol, only messages newly generated by the source node are allowed into the queue after the queue is full, while messages transmitted by other sensors are definitely dropped. The performance metrics we used in our simulations are:

**Data delivery ratio**, which is the ratio of the data received by the sink node to the sum of data generated by all the sensors in the network.
**Data delivery delay**, which is defined as the duration from the very beginning of data generation until it is received by the sink node.
**Data delivery overhead**, Energy consumption of sensors is due mainly to data transmission. Thus, the more duplicated copies generated, the higher the data delivery overhead.
**Network lifetime**, which is defined as the duration from the very beginning of the network operation until a half of sensor nodes die in our simulation.

All the simulation results are averaged over 100,000 independent runs.

### 5.2. The Geographic Locations of Nodes at Different Time

**Figure 4.**
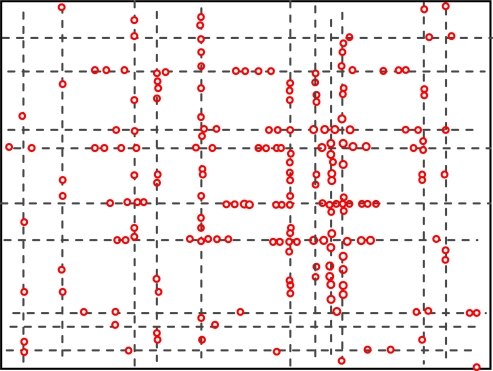
Node distributions at 8 AM.

**Figure 5.**
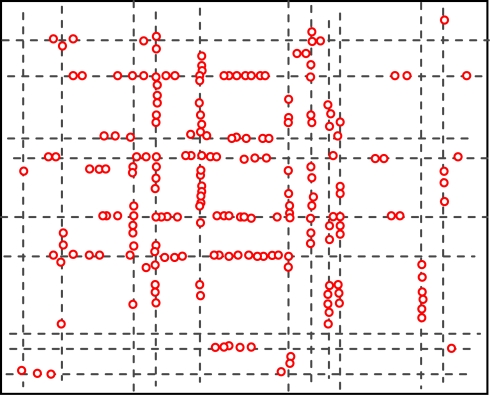
Node distributions at 12 AM.

Figures 4 and 5 show the geographic locations of nodes at 8 AM and 12 AM respectively. We can see that nodes change their locations.

The overall node distribution, while different at these different moments, remains random in general because all different types of addresses are randomly distributed in the simulation area. We can also see that more nodes occupy the central of the area than the boundary part, which indicate that nodes have a tendency to move towards the center. This phenomenon has already been addressed by many researchers. However, when the realistic situation is concerned, a non-uniform distribution might appear, e.g., homes have more population at night.

### 5.3. Performance Comparison

We compare the performance of the three protocols under the default parameters, with the results presented in Table 4.

As we can seen, the ARP protocol achieves the highest delivery ratio with lower transmission overhead (indicated by the average copies for each message) and the lowest average delivery delay, compared with other two protocols. The LBEL scheme performs worst in terms of delivery ratio. This is reasonable because the sensor nodes here only transmit messages to the node belonging to the same group (same label) as the destinations. It is ineffective for message delivery in the network. Moreover, we observe that for the flooding protocol, the delivery ratio is higher and its average delay is much longer than that of the LABEL scheme. This stems from the queue management of flooding where only messages newly generated by the source sensor are allowed into the queue after the queue is full. Accordingly, messages in the data queue can be divided into two types: message copies created by the flooding stage and single data messages coming afterwards. The former can be delivered to the sink node by different sensor nodes, while the latter can be delivered to sink nodes by the source node only. As a result, the overall delivery ratio of flooding protocol outperforms the LABEL scheme. Since the single data messages can only be transmitted to the sink nodes by the source sensor node, its average delay is much longer.

**Figure 6.**
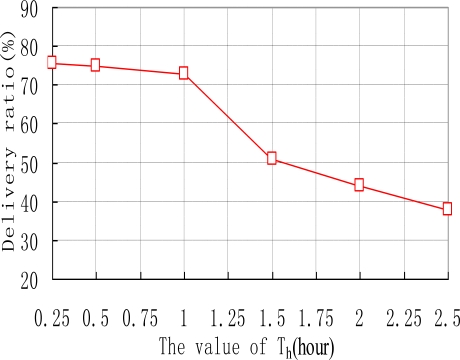
data delivery *vs.* value of *Th.*

**Figure 7.**
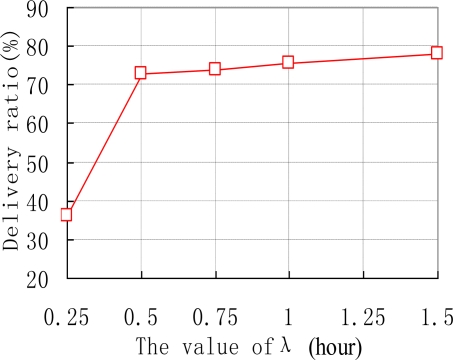
Data delivery *vs.* value of λ.

We also examine how the thresholds of Th and λ affect the performance of ARP. e.g., the data delivery ratios of ARP with different values of Th are shown in Figure 6. As we can see, the delivery ratio of ARP decreases with the increase of Th. This is natural as upgrading threshold Th for too long makes sensor nodes update their transmission ranking at quite a slow speed. Thus sensor nodes’ transmission rankings will be inaccurate, resulting in missed transmissions in the network. Figure 7 depicts how different values of λ affect the performance of ARP. We acquire that with the increase of λ, the data delivery ratio increases. This is because with too small value a of λ, the time interval that a sensor node uses to reduce its transmission ranking is improperly short. Therefore, transmission rankings for sensor nodes would be unreasonable and they may not accurately reflect the sensor nodes’ communication strength with sink nodes. As data transmission is based on the transmission rankings of sensor nodes, thus the delivery ratio is relatively small.

### 5.4. Impact of Varying Sensor Node Density

**Figure 8.**
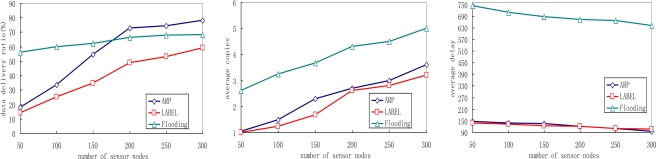
Impact of sensor node density: **(a)** Average delivery ratio; **(b)** Average copies; **(c)** Average delay.

Figure 8 illustrates the impact of sensor node density by varying the total number of people equipped with sensor nodes in the network.

We see that the ARP scheme outperforms the other two protocols in terms of delivery ratio. With the increase of sensor node density, the delivery ratios of all three schemes increase. This is reasonable since the number of neighboring sensors of each sensor node increases, and more sensor nodes help relaying messages and messages have a better chance of reaching sink nodes. Meanwhile, we also notice that flooding protocol achieves the highest data delivery ratio when a low node density is deployed. This can be explained as follows: the number of neighboring sensors for each sensor node decreases when fewer nodes are deployed, thus fewer message copies are generated in the network. Since the buffer queue may be large enough for these copies to fit in, the data delivery ratio increases as a result. Figure 8(b) shows that the number of average copies increases in all protocols with higher node density, because the number of neighboring sensors of each sensor node increase then. As more copies can increase the opportunity to deliver the matched message to sink nodes, the average data delivery delay decreases in ARP, LABEL and flooding protocols with the increase of sensor node density.

### 5.5. Impact of Varying Queue Length

**Figure 9.**
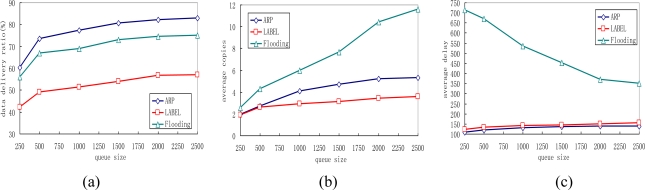
Impact of queue length: **(a)** Average delivery ratio; **(b)** Average copies; **(c)** Average delay.

We also vary the queue length of sensor nodes in our simulations, with the results presented in Figure 9. The queue length here indicates the maximum messages the sensor can hold. With an increase of queue length, the delivery ratio increases for all protocols because messages can then stay in the memory for a longer time before they are dropped. It is also noticed that ARP achieves higher gain than other protocols with the increase of queue length. As shown in Figure 9(b), with the increase of maximum queue length, the message copies in all protocols rise. This is reasonable for more copies can be accomodated in the queue before being dropped when the queue length is large. However, the ARP can always control well its transmission overhead even when the available queue size is small. Figure 9(c) depicts that the average data delivery delay decreases with larger queue length while the delivery delay in the flooding protocol is more sensitive to the variation of the queue length.

### 5.6. Impact of Varying the Number of Sink Nodes

**Figure 10.**
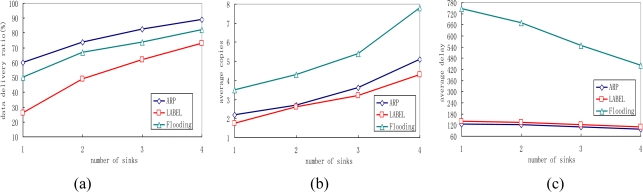
Impact of the number of sink nodes: **(a)** Average delivery ratio; **(b)** Average copies; **(c)** Average delay.

Network performance is closely related to the number of sink nodes in the network, so this group of experiments analyses performance of the three different protocols by varying the total number of sinks. The experimental results can be seen in Figure 10.

We can easily verify that as the number of sink nodes increases, the delivery ratios of all protocols rise and ARP always achieves the best performance compared with the other two protocols. This is because sensor nodes have better opportunity of meeting sink nodes as the number of sink nodes increases. Thus, messages have a better chance to be delivered before they are dropped. We also find in the experiment that the locations where the sink nodes put have great impact on data delivery ratio. If the sink node is put near “office” marked as 3, a data delivery ratio of 60% can be achieved, but if the sink node is put near “office” named 3, the data delivery ratio can only reach 46.2%.

It is also noticed that the transmission overhead of the proposed ARP, LABEL and flooding protocols all increase with the increase of the number of sink nodes, as shown in Figure 10(b). Additionally, the number of average message copies generated in the flooding protocol is much larger than that in both ARP and LABEL. This is also steme from the queue management scheme in flooding. Figure 10(c) depicts the delivery delay of all protocols decrease when the sink nodes in the network increase. This is reasonable because messages have higher opportunity of meeting the sink nodes.

### 5.7. Network Life Analysis

Network life is a very important metric for DTMSNs, because the sensor nodes are generally energy-constrained. We assume the network ends when half of the sensor nodes exhaust their energy. The initial energy reserve of each sensor node is 10 J. The simulation results are presented in Table 5.

We can see that the LABEL scheme enjoys the longest network lifetime, since sensors receive and transmit messages to destinations, or to nodes belonging to the same group as the destinations only. The flooding protocol has the shortest network lifetime. This is because too many message copies are generated and these copies consume much sensor energy. We also see that ARP achieves a longer network lifetime than flooding, which is mainly attributed to both its data delivery scheme and the effectiveness of the data delivery scheme. Though the network lifetime of ARP is shorter than LABEL, the data delivery ratio of ARP is much higher than that in LABEL scheme, demonstrating that the proposed ARP scheme can better deal with the tradeoff between the data delivery ratio and the delivery overhead than LABEL.

## 6. Conclusions

Most DTMSN routing protocols use simplistic random models for system design and evaluation, while these random models cannot reflect the real characteristics of human mobility. This paper focuses on routing for mobile human environments. Capturing the influence of peoples’ social roles, we propose ARP, a social characteristics-based routing scheme. Simulations have been carried out for performance evaluation. The results show that, compared with other DTMSN routing approaches, the proposed ARP protocol achieves a higher message delivery ratio with lower delay and transmission overhead. In the future, we plan to continue our research in the following directions: firstly, we will refine the ARP scheme by considering a more careful selection of potential relay nodes. Secondly, real experimental human mobility data will be used in simulation.

## Figures and Tables

**Table 1.** Node characteristics in five mobility models.

**Mobility Model**	**Location preference**	**Realistic**	**Group-based**	**Independent of each other**

Random Walk Model	No	No	No	Yes
Random Waypoint Model	No	No	No	Yes
Column Mobility Model	No	No	Yes	No
Nomadic Community Mobility	No	No	Yes	No
Community-based Model	Yes	Yes	No	Yes
Agenda Model	Yes	Yes	No	Yes

**Table 2.** The contact table of a sensor node.

**Transmission ranking**	**Contact duration**
1	A1
2	A2
⋮	⋮

**Table 3.** Simulation parameters.

**Parameter**	**Default Value**
Number of sensor node	200
Number of sink node	2
Initial energy of each sensor node (J)	10 J
Size of each messages(bite)	200 bits
Maximum queue size of sensor	500
Message generate interval	20 min
Value of T_h_	60 min
Value of λ	30 min
Maximum delay tolerant value β	200 min
E *_elec_*	50 nJ/bit
ɛ__fs_	10 pJ/bit/m^2^
ɛ__mp_	0.0013 pJ/bit/m^4^

**Table 4.** Simulation results with default parameters.

	**ARP**	**LABEL**	**Flooding**
**Delivery ratio (%)**	73.5	49̃0	66.8
**Average copies for each message**	2.7	2.6	4.3
**Average delay(s)**	120.5	134.2	671.2

**Table 5.** Network life with default parameters.

	**ARP**	**LABEL**	**Flooding**
Network lifetime (days)	8.48	25.2	5.69
Data delivery ratio (%)	73.5	49.0	66.8
